# Nedd8 processing enzymes in *Schizosaccharomyces pombe*

**DOI:** 10.1186/1471-2091-14-8

**Published:** 2013-03-15

**Authors:** Jean E O’Donoghue, Dawadschargal Bech-Otschir, Ida B Larsen, Mairi Wallace, Rasmus Hartmann-Petersen, Colin Gordon

**Affiliations:** 1 MRC Human Genetics Unit, Western General Hospital, Crewe Road, Edinburgh, EH4 2XU, UK; 2 Department of Biology, University of Copenhagen, Ole Maaløes Vej 5, Copenhagen, DK-2200, Denmark

**Keywords:** Ubiquitin, Nedd8, Rub1, Cullin, Protein degradation, Precursor processing

## Abstract

**Background:**

Conjugation of the ubiquitin-like modifier Nedd8 to cullins is critical for the function of SCF-type ubiquitin ligases and thus facilitates ubiquitin conjugation and ultimately degradation of SCF substrates, including several cell cycle regulators. Like ubiquitin, Nedd8 is produced as a precursor that must first be processed before it becomes active. In *Saccharomyces cerevisiae* this is carried out exclusively by the enzyme Yuh1.

**Results:**

Here we show that in the fission yeast, *Schizosaccharomyces pombe*, the Yuh1 orthologue, Uch1, is not the sole Nedd8 processing enzyme. Instead it appears that deubiquitylating enzymes can efficiently process the Nedd8 precursor *in vivo*.

**Conclusions:**

Several enzymes contribute to Nedd8 precursor processing including a number of deubiquitylating enzymes.

## Background

Post translational modification of proteins with ubiquitin and ubiquitin-like modifiers is an essential mechanism that regulates many cellular processes including protein degradation, endocytosis, DNA repair and cell signalling. The protein called Nedd8 in humans and Rub1 in budding yeast is a ubiquitin-like modifier that is covalently conjugated to a lysine residue in the cullin subunits of SCF-type E3 ubiquitin-protein ligases [1] and possibly a few other proteins [2,3]. The Nedd8 modification, also known as neddylation, of cullins is important for function and the recruitment of E2s and other components [4-6] to SCF-type ubiquitin ligases and therefore facilitates ubiquitin conjugation of SCF substrates. Similar to ubiquitin, Nedd8 conjugation requires an E1, E2 and E3 enzyme [3]. In the fission yeast *Schizosaccharomyces pombe*[6,7], as in mammals [8], the null mutation of either Nedd8 itself or components of its conjugation pathway, *i.e.* its E1 or E2 enzymes, are lethal to the cell. Curiously, the Nedd8 system is not essential for viability of the budding yeast *Saccharomyces cerevisiae*[9,10]. The reason for this difference is unknown, but could reflect differences in Nedd8 substrates between these organisms.

Just as ubiquitylation is reversed by ubiquitin isopeptidases, Nedd8 conjugation is reversed by Nedd8 isopeptidases in a process known as deneddylation. The best characterized deneddylating enzymes include the COP9 signalosome subunit Csn5 [11-13] and NEDP1 (also known as SENP8 and DEN1) [14,15]. In general these enzymes are surprisingly specific for Nedd8 over ubiquitin [3,14], but some have been shown to have dual specificity [15,16]. More recently, certain cysteine proteases, encoded by herpesviruses, were also shown to display deneddylase activity [17].

In all eukaryotic organisms the *NEDD8* gene is conserved (Additional file 1: Figure S1) and encodes a non-conjugatable precursor that contains one or more residues downstream of the mature C-terminus at position 76. To generate mature Nedd8 from the inactive precursor, specific hydrolases cleave off the C-terminal residues generating a diglycine motif in the mature Nedd8 C-terminus [1]. This precursor processing is strictly required for Nedd8’s recognition by the E1 enzyme and so the enzymes that catalyse this processing must be essential for Nedd8 function.

In *S. cerevisiae* the sole Nedd8-precursor processing enzyme has been identified as Yuh1 [18] (*S. pombe* Uch1, Additional file 1: Figure S2). In the Yuh1 null mutant, there is no longer any neddylation of cullin1 [18]. Here, we present results aimed at identifying the Nedd8 processing enzyme(s) important for Nedd8 function in *S. pombe*. We show that unlike the situation in budding yeast, *S. pombe* cells contain several enzymes capable of processing the Nedd8 precursor.

## Methods

### S. pombe strains and techniques

Fission yeast strains used in this study are derivatives of the wild type heterothallic strains *972h*^*-*^ and *975h*^*+*^. Standard genetic methods and media were used and *S. pombe* transformations were performed using the lithium acetate procedure [19]. The PCR mutagenesis was performed according to a previously published procedure [20].

### Antibodies

The antibody to tubulin was the TAT-1 monoclonal (Sigma). The antibodies to *S. pombe* Cul1/Pcu1 have been described before [21], and were kindly provided by Prof. Dieter A. Wolf (La Jolla, USA).

### Plasmids, expression and purification

The *E. coli* expression constructs used here were wild type cDNA encoding Uch1, Uch2, Nep1 and Nep2 subcloned to the pGEX-KG or pGEX-6p-1 vector (GE Healthcare). All recombinant proteins were expressed as glutathione S-transferase (GST) fusion proteins in *E. coli* BL21 (DE3) pLysS and purified on glutathione-Sepharose beads (GE Healthcare) by standard methods. The protein: bead ratio was normalized by SDS-PAGE and Coomassie staining.

### Assays

For analyses of the deneddylating activity of purified recombinant proteins and in cell extracts, the flourogenic substrate, Nedd8-7-amino-4-methylcoumarin (Nedd8-AMC) (Boston Biochemicals) was used according the instructions provided by the manufacturer and as described previously [22]. Protein concentrations were determined by Bradford assays (Pierce). Ubiquitin aldehyde (Enzo Life Sciences) was used at 5 μM, while o-phenanthroline (Sigma) was used at 5 mM.

## Results

### Uch1/Yuh1 is a deneddylating enzyme

The orthologue of *YUH1* in *S. pombe* is *uch1*^+^, which has a paralogue, *uch2*^+^. To investigate the role of *uch1*^+^ in the Nedd8 pathway, we created a null mutation in the *uch1*^+^ gene by PCR mutagenesis.

**Figure 1:**
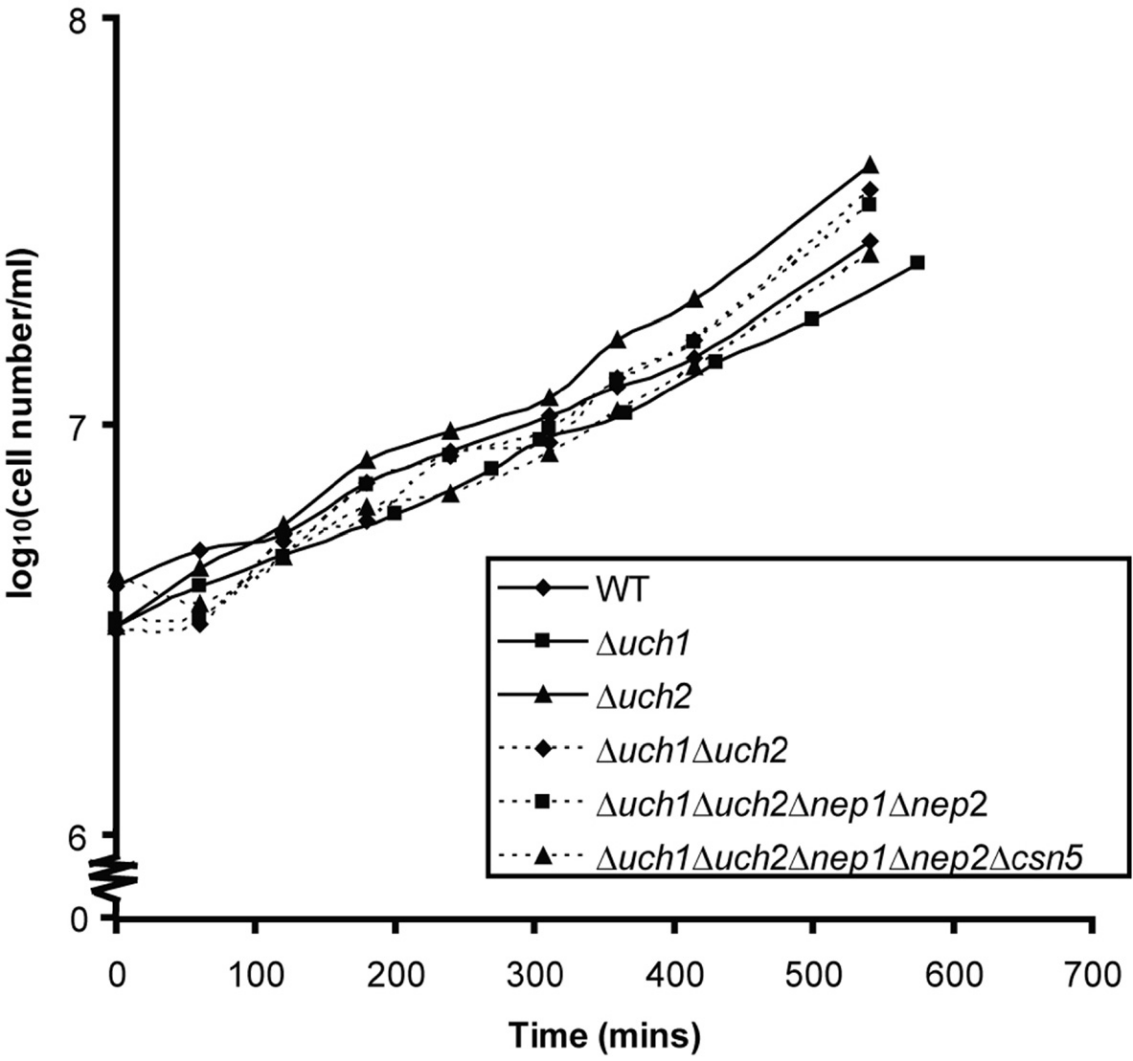
**Growth curves of wild type (WT) and mutant strains.** All mutant strains were viable and showed growth characteristics similar to the WT strain. The doubling time was about 2 hours and 45 minutes.

**Figure 2:**
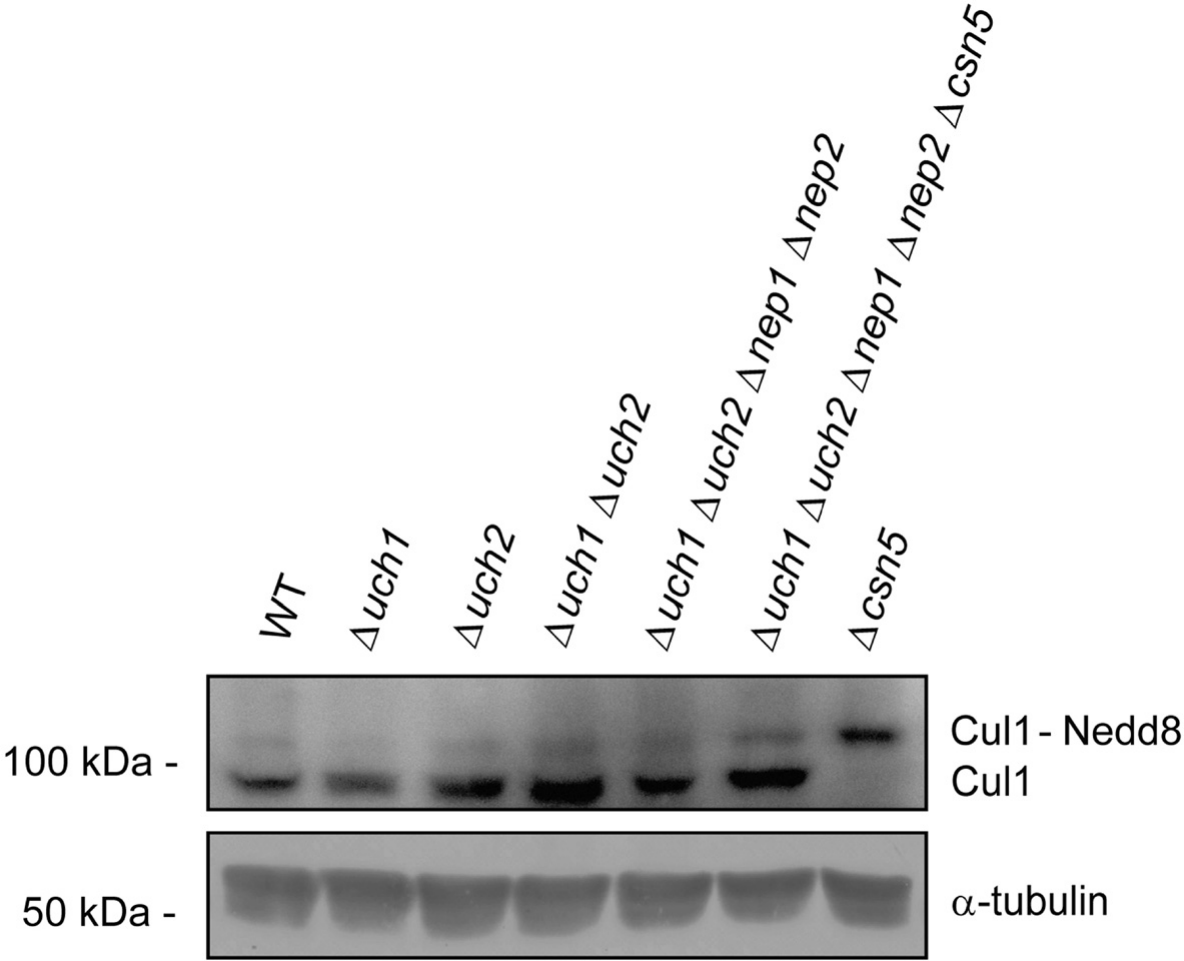
**SDS-PAGE and western blotting showing the neddylation status of Cul1 in the wild type (WT) and mutant strains.** In all strains a band corresponding to a neddylated Cul1 can be detected using an anti-Cul1 antibody. Tubulin was used to check for equal loading.

We first noted that cells carrying this *Δuch1::G418* mutation were viable (Figure 1) and appeared to have no physiological ill effects. If Uch1, as Yuh1 in *S. cerevisiae*, was the sole Nedd8-precursor processing enzyme, we would have expected the *uch1*^+^ deletion to be lethal. In order to detect any change in the neddylation status of a Nedd8 substrate we replicated the experiment carried out in *S. cerevisiae*[18]. We used an antibody to fission yeast cullin 1 (Cul1/Pcu1) [21] to detect Cul1 in cell extracts prepared from the *Δuch1* strain. Unlike in *S. cerevisiae*, Cul1 was neddylated to a similar extent in wild type and *Δuch1 S. pombe* strains (Figure 2).

**Figure 3:**
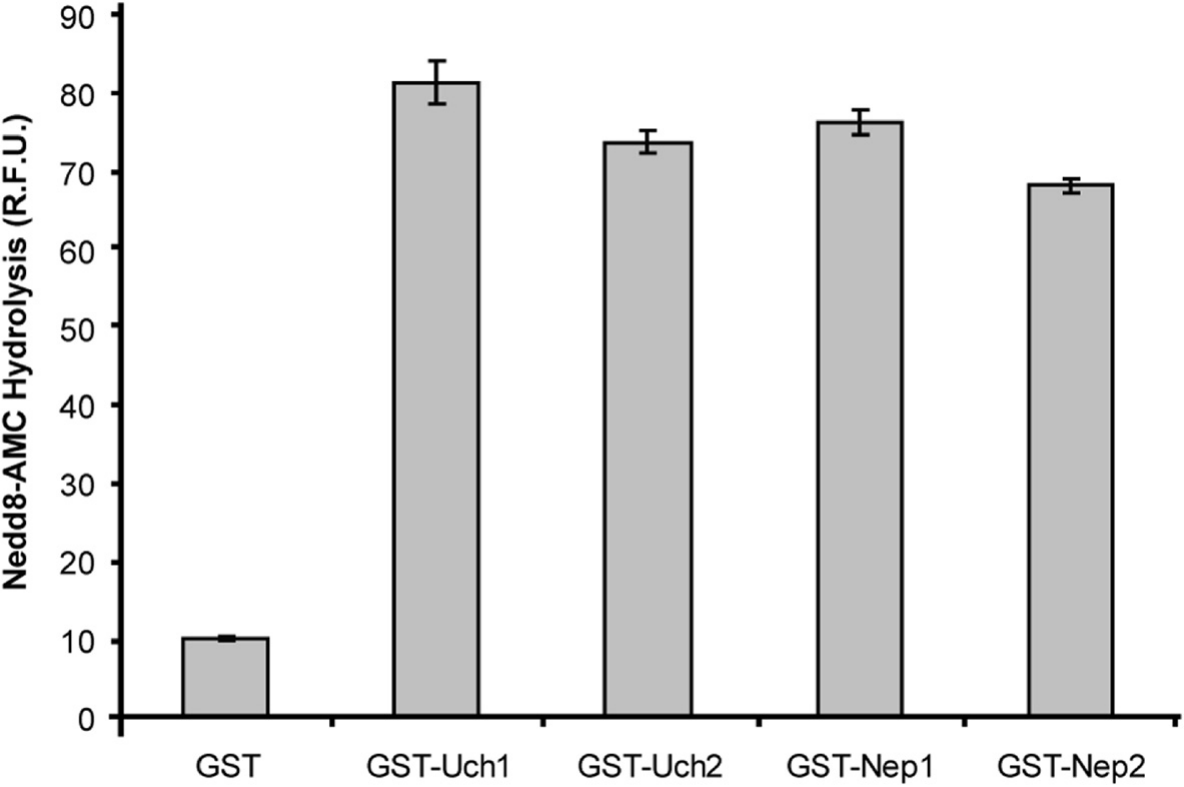
**Nedd8-AMC processing activity of Uch1, Uch2, Nep1 and Nep2.** While incubation with GST alone does not result in cleavage of Nedd8-AMC, GST-Uch1, GST-Uch2, GST-Nep1 and GST-Nep2 can all cleave Nedd8-AMC (n = 5, S.E.M. shown as error bars).

To assess whether or not *S. pombe* Uch1 could process Nedd8-precursor *in vitro* we purified a GST-tagged version of Uch1 and used Nedd8-AMC as a substrate. From this, we observed that Uch1 was indeed capable of processing Nedd8-AMC *in vitro* (Figure 3).

### Uch2/Uch37 accounts for some deneddylating activity in fission yeast

In an attempt to isolate other potential Nedd8-precursor processing enzymes we turned to *uch2*^+^, a paralogue of *uch1*^+^, and carried out the same set of experiments as described above using a GST-tagged version of Uch2 (Figure 3), a *Δuch2::ura4* strain, and a double *Δuch1Δuch2* strain (Figures 1– 2). As can be seen from these data, despite the fact that Uch2 is capable of processing Nedd8-AMC *in vitro* (Figure 3), the deletion of both homologues of *YUH1* in *S. pombe* does not result in any loss of Cul1 neddylation (Figure 2). In addition, no loss in cell viability was apparent (Figure 1), as would be expected in a Nedd8 compromised *S. pombe* strain*.*

### Deneddylating enzymes are functionally redundant

Two genes that appear to be orthologues of the mammalian NEDP1/DEN1 have been identified in *S. pombe*[23]. These are *nep1*^+^ and *nep2*^+^. In mammalian cells NEDP1/DEN1 has been described as a Nedd8-precursor processing enzyme [24], and while *S. pombe* has two such orthologues, *S. cerevisiae* has none. We hypothesised therefore that these enzymes may possess the remaining Nedd8-precursor processing activity in *S. pombe* that was unaccounted for by the two *YUH1* orthologues. To ascertain this, we created GST-tagged versions of Nep1 and Nep2 and showed that these have processing activity *in vitro* (Figure 3). We then created null mutants in each of these genes, *Δnep1::G418* and *Δnep2::arg3*, and crossed them to our double mutant *Δuch1Δuch2* strain in attempt to completely abrogate processing function in fission yeast. Surprisingly, we found that this *Δnep1Δnep2Δuch1Δuch2* strain was entirely viable (Figure 1), and Cul1 could be efficiently neddylated in this strain (Figure 2).

The final candidate we examined as a potential Nedd8 processing enzyme in fission yeast was *csn5*^+^. This subunit of the COP9/signalosome complex is well described as a metalloprotease capable of deconjugating Nedd8 from its cullin substrates [25,26]. Indeed, as observed before [21,26], hyperneddylation of Nedd8 was readily apparent in our *Δcsn5::ura4* mutant (Figure 2). Whether Csn5 can process Nedd8-precursor, however, is unclear. The *Δcsn5* mutant was crossed to the quadruple knockout strain to create a *Δnep1Δnep2Δuch1Δuch2Δcsn5* strain. Even this quintuple knockout strain was viable (Figure 1). However, the hyperneddylation of Cul1 observed in the *Δcsn5* single mutant (Figure 2) was not as apparent in the *Δnep1Δnep2Δuch1Δuch2Δcsn5* strain, suggesting that a reduced amount of processed Nedd8 is available for Cul1 modification in this background (Figure 2). Despite this effect on Cul1 neddylation, the quintuple knockout strain did not display any growth defect when compared to the wild type control or the other mutants (Figure 1).

### Deubiquitylating enzymes also provide deneddylating activity

**Figure 4:**
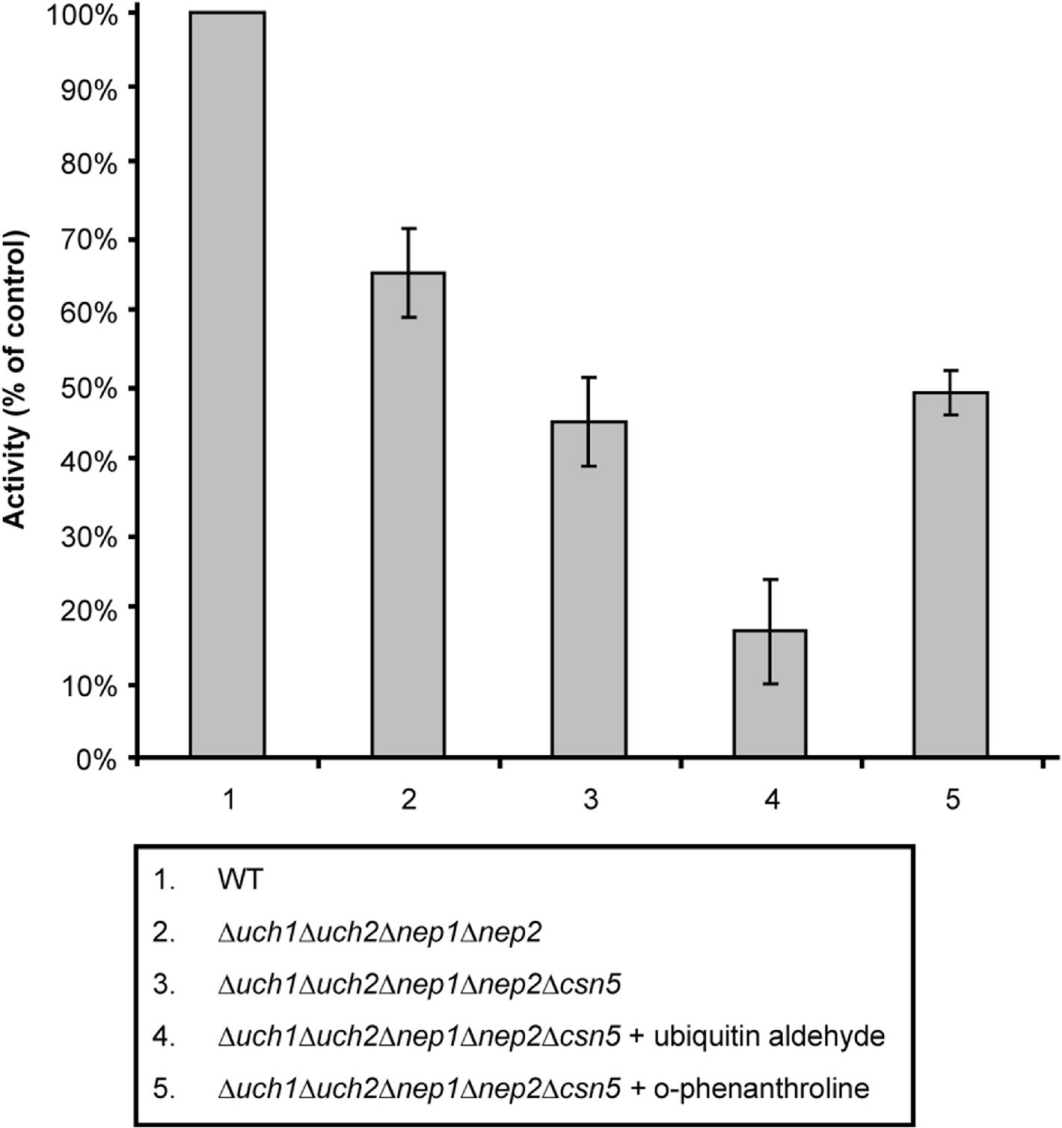
**Nedd8-AMC processing ability of some of the mutant strains.** The *Δuch1Δuch2Δnep1Δnep2* strain shows reduced levels of activity (66% of wild type) while the 5 gene deletion strain, *Δuch1Δuch2Δnep1Δnep2Δcsn5* shows even less activity (45% of wild type). However there is still processing activity unaccounted for by these 5 enzymes. This activity can be further reduced upon incubation with ubiquitin-aldehyde, but not o-phenanthroline. This suggests that other ubiquitin proteases could account for the remaining activity, but that these proteases are not metalloproteases. (n = 5, S.E.M. shown as error bars).

In an attempt to estimate the remaining Nedd8-precursor processing activity we examined the Nedd8-AMC processing activity of the *Δnep1Δnep2Δuch1Δuch2* and *Δnep1Δnep2Δuch1Δuch2Δcsn5* strains. This was done by preparing protein extracts of the strains and incubating with Nedd8-AMC as described previously [22]. The results revealed that albeit the processing activity in these strains was reduced, it was by no means eliminated (Figure 4). Most deubiquitylating and deneddylating enzymes in fission yeast are cysteine proteases, but a few are metalloproteases [27]. To determine what may be responsible for this remaining activity in the quintuple mutant we used two enzymatic inhibitors, ubiquitin aldehyde and o-phenanthroline. Ubiquitin aldehyde is used to inhibit deubiquitylating enzymes, while o-phenanthroline inhibits metalloproteases. As evident from the data, while ubiquitin aldehyde can inhibit some of the remaining activity from the *Δnep1Δnep2Δuch1Δuch2Δcsn5* strain, o-phenanthroline cannot (Figure 4). This implies that the remaining activity present in the *Δnep1Δnep2Δuch1Δuch2Δcsn5* strain is due to the activity of ubiquitin protease(s), but not metalloproteases.

## Discussion

In the present work we found that unlike in *S. cerevisiae*, the Nedd8-precursor processing activity in *S. pombe* is not solely associated with the *YUH1* orthologue *uch1*^+^. In fact the deletion of five candidate genes for Nedd8-precursor processing activity appears to have little physiological effect on the cell, despite the predicted lethality associated with the lack of Nedd8-precursor processing in fission yeast.

At first we were surprised by these results, but perhaps it is not so unexpected given what we know of Nedd8 in *S. pombe*, *i.e.* that lack of Nedd8-precursor processing will undoubtedly lead to death of the cell, and that Nedd8 and ubiquitin are 50% identical in amino acid sequence. Perhaps the requirement for Nedd8-precursor processing, forces the cell to utilize those enzymes which perform a similar function for ubiquitin, turning them into Nedd8-precursor processing enzymes. This is suggested by the remaining processing activity and cell viability once the genes for the five enzymes Uch1, Uch2, Nep1, Nep2 and Csn5 were deleted. Despite these data, it is possible that in the wild type cell under normal physiological conditions, there is only one main Nedd8-precursor processing enzyme in *S. pombe*, and it is only when the function of this enzyme is lost that the cell recruits other enzymes to perform the vital task of Nedd8-precursor processing in fission yeast.

The observation that hyper neddylation of Cul1 is lost in the quintuple mutant also suggests that these five enzymes provide the cell with most of the Nedd8-precursor processing activity. However, since the quintuple mutant is viable, a limiting amount of processed Nedd8 must be sufficient to support neddylation.

Some DUBs are regulated on the level of their subcellular localization [28]. We note that our deneddylation assays were performed in cell extracts, and therefore do not take this into account.

Recently, Nedd8 overexpression was found to result in neddylation of ubiquitin substrates catalyzed by ubiquitin enzymes [29,30]. This atypical neddylation suggests that ubiquitin activating enzymes can moonlight as Nedd8 activating enzymes. The results presented here suggest that deubiquitylating enzymes similarly may also target Nedd8. These results are also in agreement with a recent paper on budding yeast Nedd8 (Rub1) cleavage [31], which shows that heterologous Rub1-ubiquitin chains are disassembled by the COP9 signalosome and proteasome-associated DUBs. Hence, although Yuh1 in budding yeast appears to be the major processing enzyme in *Saccharomyces cerevisiae*, the profound structural similarity between ubiquitin and Nedd8 [31] leads to functional cross-talk in both yeasts.

## Conclusions

Unlike the situation in budding yeast, several enzymes contribute to Nedd8 precursor processing in *S. pombe*, including a number of deubiquitylating enzymes. This functional redundancy may be connected with the essential nature of Nedd8 conjugation in fission yeast.

## Abbreviations

AMC: 7-amino-4-methylcoumarin; GST: Glutathione S-transferase; S.E.M.: Standard error of mean; WT: Wild type.

## Competing interests

The authors declare that they have no competing interests.

## Authors’ contributions

JOD, DBO, IBL and MW carried out experiments. CG and RHP conceived the study. JOD and RHP drafted the manuscript. All authors have read and approved the final manuscript.

## Supplementary Material

Additional file 1 Supporting Information. Click here for file

## References

[B1] Kamitani T Kito K Nguyen HP Yeh ET Characterization of NEDD8, a developmentally down-regulated ubiquitin-like protein J Biol Chem 1997 272 28557 28562 10.1074/jbc.272.45.28557 9353319

[B2] Xirodimas DP Sundqvist A Nakamura A Shen L Botting C Hay RT Ribosomal proteins are targets for the NEDD8 pathway EMBO Rep 2008 9 280 286 10.1038/embor.2008.10 18274552 PMC2267383

[B3] Rabut G Peter M Function and regulation of protein neddylation. 'Protein modifications: beyond the usual suspects' review series EMBO Rep 2008 9 969 976 10.1038/embor.2008.183 18802447 PMC2572130

[B4] Besten W Verma R Kleiger G Oania RS Deshaies RJ NEDD8 links cullin-RING ubiquitin ligase function to the p97 pathway Nat Struct Mol Biol 2012 19 511 516 10.1038/nsmb.2269 22466964 PMC3348432

[B5] Bandau S Knebel A Gage ZO Wood NT Alexandru G UBXN7 docks on neddylated cullin complexes using its UIM motif and causes HIF1alpha accumulation BMC Biol 2012 10 36 10.1186/1741-7007-10-36 22537386 PMC3349548

[B6] Osaka F Saeki M Katayama S Aida N Toh E Kominami K Toda T Suzuki T Chiba T Tanaka K Kato S Covalent modifier NEDD8 is essential for SCF ubiquitin-ligase in fission yeast EMBO J 2000 19 3475 3484 10.1093/emboj/19.13.3475 10880460 PMC313942

[B7] Girdwood D Xirodimas DP Gordon C The essential functions of NEDD8 are mediated via distinct surface regions, and not by polyneddylation in Schizosaccharomyces pombe PLoS One 2011 6 e20089 10.1371/journal.pone.0020089 21655279 PMC3105002

[B8] Tateishi K Omata M Tanaka K Chiba T The NEDD8 system is essential for cell cycle progression and morphogenetic pathway in mice J Cell Biol 2001 155 571 579 10.1083/jcb.200104035 11696557 PMC2198877

[B9] Lammer D Mathias N Laplaza JM Jiang W Liu Y Callis J Goebl M Estelle M Modification of yeast Cdc53p by the ubiquitin-related protein rub1p affects function of the SCFCdc4 complex Genes Dev 1998 12 914 926 10.1101/gad.12.7.914 9531531 PMC316682

[B10] Liakopoulos D Doenges G Matuschewski K Jentsch S A novel protein modification pathway related to the ubiquitin system EMBO J 1998 17 2208 2214 10.1093/emboj/17.8.2208 9545234 PMC1170565

[B11] Schwechheimer C The COP9 signalosome (CSN): an evolutionary conserved proteolysis regulator in eukaryotic development Biochim Biophys Acta 2004 1695 45 54 10.1016/j.bbamcr.2004.09.023 15571808

[B12] Cope GA Deshaies RJ COP9 signalosome: a multifunctional regulator of SCF and other cullin-based ubiquitin ligases Cell 2003 114 663 671 10.1016/S0092-8674(03)00722-0 14505567

[B13] Schmaler T Dubiel W Control of Deneddylation by the COP9 Signalosome Subcell Biochem 2010 54 57 68 10.1007/978-1-4419-6676-6_5 21222273

[B14] Shen LN Liu H Dong C Xirodimas D Naismith JH Hay RT Structural basis of NEDD8 ubiquitin discrimination by the deNEDDylating enzyme NEDP1 EMBO J 2005 24 1341 1351 10.1038/sj.emboj.7600628 15775960 PMC1142549

[B15] Gan-Erdene T Nagamalleswari K Yin L Wu K Pan ZQ Wilkinson KD Identification and characterization of DEN1, a deneddylase of the ULP family J Biol Chem 2003 278 28892 28900 10.1074/jbc.M302890200 12759362

[B16] Hemelaar J Borodovsky A Kessler BM Reverter D Cook J Kolli N Gan-Erdene T Wilkinson KD Gill G Lima CD Ploegh HL Ovaa H Specific and covalent targeting of conjugating and deconjugating enzymes of ubiquitin-like proteins Mol Cell Biol 2004 24 84 95 10.1128/MCB.24.1.84-95.2004 14673145 PMC303361

[B17] Gastaldello S Hildebrand S Faridani O Callegari S Palmkvist M Di GC Masucci MG A deneddylase encoded by Epstein-Barr virus promotes viral DNA replication by regulating the activity of cullin-RING ligases Nat Cell Biol 2010 12 351 361 10.1038/ncb2035 20190741

[B18] Linghu B Callis J Goebl MG Rub1p processing by Yuh1p is required for wild-type levels of Rub1p conjugation to Cdc53p Eukaryot Cell 2002 1 491 494 10.1128/EC.1.3.491-494.2002 12455997 PMC118023

[B19] Moreno S Klar A Nurse P Molecular genetic analysis of fission yeast Schizosaccharomyces pombe Methods Enzymol 1991 194 795 823 2005825 10.1016/0076-6879(91)94059-l

[B20] Bahler J Wu JQ Longtine MS Shah NG McKenzie A III Steever AB Wach A Philippsen P Pringle JR Heterologous modules for efficient and versatile PCR-based gene targeting in Schizosaccharomyces pombe Yeast 1998 14 943 951 10.1002/(SICI)1097-0061(199807)14:10<943::AID-YEA292>3.0.CO;2-Y 9717240

[B21] Schmidt MW McQuary PR Wee S Hofmann K Wolf DA F-box-directed CRL complex assembly and regulation by the CSN and CAND1 Mol Cell 2009 35 586 597 10.1016/j.molcel.2009.07.024 19748355 PMC2779159

[B22] Stone M Hartmann-Petersen R Seeger M Bech-Otschir D Wallace M Gordon C Uch2/Uch37 is the major deubiquitinating enzyme associated with the 26S proteasome in fission yeast J Mol Biol 2004 344 697 706 10.1016/j.jmb.2004.09.057 15533439

[B23] Zhou L Watts FZ Nep1, a Schizosaccharomyces pombe deneddylating enzyme Biochem J 2005 389 307 314 10.1042/BJ20041991 15769255 PMC1175107

[B24] Mendoza HM Shen LN Botting C Lewis A Chen J Ink B Hay RT NEDP1, a highly conserved cysteine protease that deNEDDylates Cullins J Biol Chem 2003 278 25637 25643 10.1074/jbc.M212948200 12730221

[B25] Cope GA Suh GS Aravind L Schwarz SE Zipursky SL Koonin EV Deshaies RJ Role of predicted metalloprotease motif of Jab1/Csn5 in cleavage of Nedd8 from Cul1 Science 2002 298 608 611 10.1126/science.1075901 12183637

[B26] Mundt KE Liu C Carr AM Deletion mutants in COP9/signalosome subunits in fission yeast Schizosaccharomyces pombe display distinct phenotypes Mol Biol Cell 2002 13 493 502 10.1091/mbc.01-10-0521 11854407 PMC65644

[B27] Kouranti I McLean JR Feoktistova A Liang P Johnson AE Roberts-Galbraith RH Gould KL A global census of fission yeast deubiquitinating enzyme localization and interaction networks reveals distinct compartmentalization profiles and overlapping functions in endocytosis and polarity PLoS Biol 2010 8 e1000471 10.1371/journal.pbio.1000471 20838651 PMC2935449

[B28] Reyes-Turcu FE Ventii KH Wilkinson KD Regulation and cellular roles of ubiquitin-specific deubiquitinating enzymes Annu Rev Biochem 2009 78 363 397 10.1146/annurev.biochem.78.082307.091526 19489724 PMC2734102

[B29] Hjerpe R Thomas Y Chen J Zemla A Curran S Shpiro N Dick LR Kurz T Changes in the ratio of free NEDD8 to ubiquitin triggers NEDDylation by ubiquitin enzymes Biochem J 2012 441 927 936 10.1042/BJ20111671 22004789 PMC3280039

[B30] Leidecker O Matic I Mahata B Pion E Xirodimas DP The ubiquitin E1 enzyme Ube1 mediates NEDD8 activation under diverse stress conditions Cell Cycle 2012 11 1142 1150 10.4161/cc.11.6.19559 22370482

[B31] Singh RK Zerath S Kleifeld O Scheffner M Glickman MH Fushman D Recognition and Cleavage of Rub1 and Rub1-Ubiquitin Chains by Components of the Ubiquitin-Proteasome System Mol Cell Proteomics 2012 11 1595 1611 10.1074/mcp.M112.022467 23105008 PMC3518131

