# Supplementary material for: Nedd8 processing enzymes in Schizosaccharomyces pombe
Source: BMC Biochem. 2013 Mar 15;14:8. doi: 10.1186/1471-2091-14-8 (PMC3602023; doi:10.1186/1471-2091-14-8)
Supplement: Additional file 1 — Supporting Information. [file 1471-2091-14-8-S1.pdf]

Supporting Information

```
Nedd8 Hs MLIKVKTLTGKEIEIDIEPTDKVERIKERVEEKEGIPPQQQRLIYSGKQMNDEKTAADYK 60
Nedd8 Mm MLIKVKTLTGKEIEIDIEPTDKVERIKERVEEKEGIPPQQQRLIYSGKQMNDEKTAADYK 60
Nedd8 Dm MLIKVKTLTGKEIEIDIEPTDKVDRIKERVEEKEGIPPQQQRLIFSGKQMNDDKTAADYK 60
Nedd8 Bt MLIKVKTLTGKEIEIDIEPTDKVERIKERVEEKEGIPPQQQRLIYSGKQMNDEKTAADYK 60
Rub1 Sc  MIVKVKTLTGKEISVELKESDLVYHIKELLEEKEGIPPSQQRLIFQGKQIDDKLTVTDAH 60
Ned8 Sp  MLIKVKTLTGKEIELDIDPNDKVSRIKERVEEKEGIPPSQQRLIYAGKQMADDKNAESYH 60
         *::**********.:::. .* * :*** :******** *****: ***: *. .. . :

Nedd8 Hs ILGGSVLHLVLALRGGGGLRQ--- 81
Nedd8 Mm ILGGSVLHLVLALRGGGGLGQ--- 81
Nedd8 Dm VQGGSVLHLVLALRGGDSILTPCV 84
Nedd8 Bt ILGGSVLHLVLALRGGGGLRQ--- 81
Rub1 Sc  LVEGMQLHLVLTLRGGN------- 77
Ned8 Sp  LEGGSVLHLVLALRGGSC------ 78
         :  *  *****:****
                        ↑
```

Figure S1 *Nedd8 is a phylogenetically conserved ubiquitin-like modifier*

Nedd8 from the indicated species were aligned using the ClustalW program. Similar (:) and identical (*) residues have been marked. Note that the mature C-terminal GG motif (arrow) is completely conserved.

```
Yuh1 Sc MSGENRAVVPIESNPEVFTNFAHKLGLKNEWAYFDIYSLTEPELLAFLPRPVKAIVLLFP 60
Uch1 Sp ------MWRPLENTPEVLEPYLQKIGVQ-DASVFDLFSLEE--IPEYIPRPVHALLFVFP 51
                 *:*..***:  : :*:*:: : : **::** *  :  ::****:*::::**

Yuh1 Sc INEDRKSSTSQQITS--SYDVIWFKQSVKNACGLYAILHSLSNNQSLLEPGSDLDNFLKS 118
Uch1 Sp SSGTKTIYKGSRILPKDSDKVLWYPQTIPNACGTIGLLHAVSNGE--LRRKVNENDFIKS 109
         .  :.  ...:* .  * .*:*: *:: ****  .:**::**.:  *.   : ::*:**

Yuh1 Sc QSDTS--SSKNRFDDVTTDQFVLNVIKENVQTFSTGQSEAPEATADTNLHYITYVE---- 172
Uch1 Sp LIRTAEGSSIEERAKLIEDSKELEALHAAFAG-PPLEVEGSEEDVETDLHFICFVKGKSK 168
               *:  ** :.  .:  *.  *:.::  .   .. : *..*  .:*:**:* :*:

Yuh1 Sc ENGGIFELDGRNLSGPLYLGKSDPTATDLIEQELVRVRVASYMEN-ANEEDVLNFAMLGL 231
Uch1 Sp DDNHFYELDGR---------QEGPVQHSEIESDLLNAEVLSVIKNYIQSIDSPFFSLVAI 219
        ::. ::*****         :..*.  . **.:*:...* * ::*  :. *   *:::.:

Yuh1 Sc GPNWE 236
Uch1 Sp TTP-- 222
```

Figure S2 *Uch1 is the fission yeast orthologue of S. cerevisiae Yuh1*

S. cerevisiae (Sc) Yuh1 and *S. pombe* (Sp) Uch1 were aligned using the ClustalW program. Similar (:) and identical (*) residues have been marked. Note that the active site cysteine residue (red) is conserved.
